# Prognostic Implications of *SMARCA4* , *ARID1A* , and Other BAF Mutations in Non‐Small Cell Lung Cancer

**DOI:** 10.1002/cam4.71442

**Published:** 2025-12-12

**Authors:** Alexis Khalil, Michael P. Collins, Alfonso Quintás‐Cardama

**Affiliations:** ^1^ Foghorn Therapeutics Inc. Cambridge Massachusetts USA

**Keywords:** BAF, BRG1, lung, NSCLC, prognosis, SMARCA4, SWI/SNF

## Abstract

**Background and Methods:**

Non‐small cell lung cancer (NSCLC) outcomes have improved remarkably with the widespread use of immune checkpoint inhibitors and small molecule inhibitors targeting driver mutations. Nevertheless, many patients continue to experience suboptimal outcomes. The prevalence of mutations in the BAF (BRG1/BRM‐associated factor) chromatin remodeling complexes may represent an opportunity to help close this gap: These critical regulators of chromatin accessibility are mutated in approximately a quarter of NSCLC cases, and numerous retrospective reports have evaluated the impact of these mutations on clinical outcomes. Here, we appraise the varying and occasionally divergent evidence for BAF complex mutations as predictive and prognostic biomarkers in NSCLC.

**Results:**

We conclude that these mutations hold promise as refinements to existing prognostic and treatment algorithms, with *SMARCA4* mutations imparting poor prognosis, *ARID1A* mutations predicting better prognosis with immune checkpoint inhibitor therapy, and *ARID1A*‐epithelial growth factor receptor (*EGFR*) comutations being associated with insensitivity to EGFR tyrosine kinase inhibitor therapy. Additional research should focus on large, prospective studies that will allow better quantification of the impact of BAF complex mutations.

**Conclusions:**

A growing body of evidence indicates that BAF complex mutations have important prognostic implications. These may be leveraged for risk stratification and therapeutic selection in patients with non‐small cell lung cancer.

## Graphical abstract

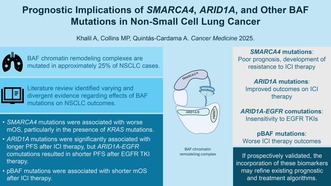

## 1 Introduction

The BAF (BRG1/BRM‐associated factor) complexes, also known as the SWI/SNF (SWItch/Sucrose Non‐Fermentable) complexes, are evolutionarily conserved multisubunit adenosine triphosphate (ATP)‐dependent chromatin remodeling complexes. Three distinct BAF complexes have been identified, namely canonical BAF (cBAF), polybromo‐associated BAF (pBAF), and non‐canonical BAF (ncBAF), which contain shared subunits and complex‐specific subunits that are combined in a tissue‐ and function‐specific manner [1]. The mutually exclusive ATP‐dependent helicases SMARCA2 and SMARCA4 (SWI/SNF‐related BAF chromatin remodeling complex subunit ATPase 2 and 4, also known as BRM and BRG1, respectively) form the core of every BAF complex, and the energy they generate via ATP hydrolysis allows BAF complexes to slide or evict nucleosomes along the DNA [1]. Multiple core and accessory subunits facilitate the binding of the complex at sequence‐specific locations, allowing sequence‐specific transcription factors and transcriptional machinery to access gene regulatory elements and control gene expression. These interactions play a key role in cellular differentiation, DNA repair, and cell cycle progression, which are critical in maintaining the pluripotency of stem cells and regulating organ development [1, 2, 3].

The rich combinatorial diversity and tissue/cell type‐specific expression of BAF subunits is likely responsible for determining which BAF complexes interact with which transcription factors, where chromatin is remodeled, and possibly why certain mutated subunits are markedly associated with specific tumor types [4, 5, 6, 7, 8]. The genes encoding subunits of the BAF complex are some of the most frequently mutated in cancer, with approximately 20% of all human cancers harboring mutations in at least one BAF subunit gene, including in highly prevalent cancers such as non‐small cell lung cancer (NSCLC) [9, 10, 11].

The impact of different BAF mutations on BAF complex assembly and function varies significantly depending on whether the mutation leads to complete or partial loss of protein expression and which subunit is mutated. Thus, the impact of different BAF mutations on chromatin remodeling and transcriptional regulation varies widely. This is best illustrated by the fact that complete loss of expression of the BAF subunits SMARCA4, ARID1A, or SMARCC1 leads to a global decrease in chromatin accessibility, while the loss of ARID1B expression is associated with the opposite effect [12]. This is important from a translational point of view, as different BAF mutational profiles thus have distinct contributions to cancer pathogenesis, resistance to therapy, and prognosis [2]. Reflecting this, a growing body of work is emerging to address the multifaceted impact of these genetic alterations in cancer prognosis.

Over the last decade, the validation of predictive biomarkers has played a critical role in allowing precise treatment selection for patients with NSCLC; clinical outcomes have markedly improved as a consequence. However, the existing biomarkers are imperfect, outcomes still vary widely, and the identification of additional predictive biomarkers remains an unmet need. Prominent among these potential new biomarkers are BAF complex mutations. Approximately 25% of NSCLC cases harbor at least one such mutation [13] and multiple studies have attempted to address their prognostic implications, sometimes with conflicting results. Not surprisingly, these studies report that prognosis is markedly influenced not only by which subunit is mutated but also by other critical factors including their interaction with other BAF mutations and/or with other oncogenes, differences in zygosity, and class of anticancer therapy. Here we summarize our current understanding regarding the impact of BAF mutations on the prognosis of patients with NSCLC.

## 2 BAF Complex Mutations

Large genomic studies have documented mutations in all subunits of the BAF complex. However, the bulk of these alterations are in genes encoding SMARCA4, ARID1A, ARID2, and PBRM1, with most localizing to subunit‐subunit interfaces, suggesting altered assembly and/or composition of BAF complexes [1]. Supporting a central role of these genes in tumorigenesis, mice genetically engineered to express inactivated *Smarca4*, *Arid1a*, *Smarcb1*, or *Pbrm1* alleles are prone to cancer [1]. The type and frequency of BAF complex mutations in humans varies substantially depending on tumor type, which suggests context‐dependent functions for specific mutated BAF subunits [3, 10]. Commonly, mutations in genes encoding subunits of the BAF complex result in complete loss of function (LoF). These mutations, referred to as class 1 [14], typically involve nonsense mutations, frameshift mutations, or large deletions. When tumor suppressor genes like *SMARCA4*, *SMARCB1*, or *ARID1A* undergo class 1 mutations, the resulting LoF renders the protein unable to regulate transcription, chromatin remodeling, cell cycle progression, and apoptosis [14], contributing to tumorigenesis. BAF subunit haploinsufficiency or complete loss thus results in the loss of a key mechanism of tumor suppression [10], and such alterations have been associated with worse prognosis across a wide range of human cancers [15]. A second set of BAF mutations, referred to as class 2 [14], includes missense and splicing mutations, which result in hypomorphic alleles and partial LoF. These types of alterations undermine the tumor suppressive function of the resulting protein to varying degrees.

In NSCLC, genetic alterations involving at least one subunit of the BAF complex have been reported in approximately 20% of patients, making them some of the most common mutations in lung cancer [16, 17]. The most frequently mutated BAF subunits in NSCLC are *SMARCA4*, *ARID1A*, *SMARCA2*, *ARID1B*, *ARID2*, *PBRM1*, and *SMARCB1* [17]. Several BAF complex‐directed therapies are currently undergoing clinical testing and some have already demonstrated clinical activity in NSCLC (Table 1).

**TABLE 1:** Agents targeting SMARCA2 and/or SMARCA4 currently in development.

Mechanism of action	Agent company	Phase (NCT #) or status	Indication	Combination or monotherapy	Preliminary safety and clinical activity results
**Clinical stage** ^**a**^					
SMARCA2 degradation	PRT3789 Prelude Therapeutics	1 (NCT05639751)	Advanced, recurrent, or metastatic solid tumors with *SMARCA4* mutation	Docetaxel	*N* = 11^b^ DLTs: 2 pts (18%) [18]
	PRT3789 Prelude Therapeutics	2 (NCT06682806)	Part 1 (safety run‐in): Advanced, recurrent, or metastatic solid tumors with *SMARCA4* mutation or SMARCA4 loss, with backfill enrichment for NSCLC with LoF *SMARCA4* mutation	Monotherapy	*N* = 69^b^ No DLTs 5 pts achieved PR (2 esophageal, 2 NSCLC, 1 gastric) [18]
			Part 2 (main study): Advanced, recurrent, or metastatic esophageal cancer or NSCLC with *SMARCA4* mutation or SMARCA4 loss	Pembrolizumab	Not available
	PRT7732 Prelude Therapeutics	1 (NCT06560645)	Advanced, recurrent, or metastatic solid tumors with *SMARCA4* mutation	Monotherapy	Not available
SMARCA2 inhibition	LY4050784 Eli Lilly and Company	1 (NCT06561685)	Phase 1a (dose escalation): Locally advanced or metastatic solid tumor with *SMARCA4* mutation	Monotherapy	Not available
			Phase 1b (expansion), Part A: Locally advanced NSCLC or metastatic NSCLC with known or likely LoF *SMARCA4* mutation or SMARCA4 loss		
			Phase 1b (expansion), Part B: Any non‐NSCLC tumor with known or likely LoF *SMARCA4* mutation or SMARCA4 loss		
**Preclinical stage**					
SMARCA2 degradation	AU‐19820/AUR‐110 Aurigene Oncology	IND approved	*SMARCA4*‐mutated cancers	Unknown	Not applicable
	PLX‐61639 Plexium	IND‐enabling	SMARCA4‐deficient solid tumors	Unknown	Not applicable

Abbreviations: AE, adverse event; DLT, dose‐limiting toxicity; IND, Investigational New Drug Application; LoF, loss of function; NCT, National Clinical Trial; NSCLC, non‐small cell lung cancer; PR, partial response; pt, patient; SAE, serious adverse event; TRAE, treatment‐related adverse event.

^a^ Based on a search of clinicaltrials.gov.

^b^ Data cutoff date: 30 November 2024.

## 3 Prognostic Relevance of *SMARCA4* Mutations

SMARCA4 is one of the core catalytic subunits of the BAF complex; SMARCA2 is its paralogous counterpart. Large genomic studies have identified *SMARCA4* mutations in approximately 4% of all cancers, with NSCLC, cancer of unknown primary, and endometrial, breast, and colon cancer having the highest prevalence [19]. Over half of the SMARCA4 mutations in human cancer samples are missense mutations that tend to cluster in the catalytic domain, at subunit–subunit interaction interfaces, and at nucleosome binding sites [1, 7]. The dysregulation of SMARCA4 results in altered transcriptional programs that increase expression of genes that foster malignant proliferation [20, 21, 22], as evidenced by nonclinical studies demonstrating that *SMARCA4* inactivation promotes the formation of aggressive and invasive tumors [23, 24]. In NSCLC, SMARCA4 alterations occur in approximately 10% of cases and have been reported in multiple studies as being among the most prognostically deleterious genomic alterations [14, 16, 19, 25] (Table 2).

**TABLE 2:** Selected studies evaluating *SMARCA4* mutations in NSCLC.

Year, author	Country	Study design	Comparison	Prognostic impact^a^	mOS (mo) HR (95% CI); *p*‐value	MVA HR (95% CI); *p*‐value
2025 Gandhi [26]	United States	Adv non‐LUSC treated with CIT; *n* = 638	Mono‐ or bi‐allelic deletion (*n* = 147, 23%)^b^ vs. WT	↓	10.8 vs. 19.0 1.7 (1.4–2.1); *p* < 0.0001	1.70 (1.33, 2.18); *p* < 0.001 (*n* = 566)
		DFCI + MSKCC *KRAS* ^WT^ cohort treated with CIT; *n* = 391	Mono‐ or bi‐allelic deletion (*n* = 96, 25%) vs. WT	↓	11.7 vs. 19.6 1.7 (1.3–2.2); *p* = 0004	Not applicable
		DFCI + MSKCC *KRAS* ^MUT^ cohort treated with CIT; *n* = 247	Mono‐ or bi‐allelic deletion (*n* = 51, 21%) vs. WT	↓	9.0 vs. 16.9 1.8 (1.2–2.7); *p* = 0.003	Not applicable
		DFCI + MSKCC cohort treated with ICI; *n* = 1099	Mono‐ or bi‐allelic deletion (*n* = 236, 22%) vs. WT	↔	13.3 vs. 14.1 1.1 (0.9–1.3); *p* = 0.31	Not applicable
		DFCI cohort treated with ICI; *n* = 616	Mono‐ or bi‐allelic deletion (*n* = 98, 16%) vs. WT	↔	13.8 vs. 13.2 1.1 (0.8–1.4); *p* = 0.66	Not applicable
		DFCI cohort not treated with ICI or CIT; *n* = 863	Mono‐ or bi‐allelic deletion (*n* = 111; 13%) vs. WT	↔	20.5 vs. 21.7 1.1 (0.9–1.4); *p* = 0.34	Not applicable
2023 Alessi [27]	United States England	Adv non‐LUSC NSCLC treated with 1st line CIT; *n* = 707	Mutation (*n* = 114, 16%) vs. WT	↓	8.1 vs. 15.0 1.70 (1.33–2.17); *p* < 0.001	1.66 (1.27–2.22); *p* < 0.001 (*n* = 618)
		*KRAS* ^WT^ cohort treated with first‐line CIT; *n* = 431	Mutation (*n* = 70, 16%) vs. WT	↔	10.0 vs. 15.0 1.36 (0.98–1.88); *p* = 0.06	Not applicable
		*KRAS* ^MUT^ cohort treated with first‐line CIT; *n* = 276	Mutation (*n* = 44, 16%) vs. WT	↓	6.6 vs. 14.6 2.52 (1.72–3.68); *p* < 0.001	
2023 Negrao [28]	France Germany Greece Italy United States	Stage IV *KRAS* ^*G12C*^‐mut NSCLC treated with sorafenib/adagrasib; *n* = 234	Mutation (*n* = 20, 9%) vs. WT	↓	4.9 vs. 11.8; *p* < 0.001 3.07 (1.69–5.60)	3.07 (1.69–5.60); *p* < 0.001
		Stage IV *KRAS* ^*G12C*^‐mut NSCLC treated with sorafenib/adagrasib; *n* = 209	Co‐mutation with *KEAP1* and *CDKN2A* mut (*n* = 81, 39%) vs. WT for all three mutations	↓	6.9 vs. 13.0; *p* < 0.001 2.05 (1.38–3.02)	Not applicable
		Stage IV *KRAS* ^*G12C*^‐mut NSCLC treated with sorafenib/adagrasib, previously treated with ICI; *n* = 183	Mutation (*n* = 15, 8%) vs. WT	↓	4.9 vs. 10.5; *p* = 0.003 2.66 (1.36–5.17)	Not applicable
2021 Alessi [16]	United States	Metastatic NSCLC (various treatments); *n* = 1490	Mutation, any deleterious (*n* = 163, 11%) vs. WT	↓	15.6 vs. 25.0 0.64 (0.53–0.78); *p* < 0.001	Not applicable
		Metastatic NSCLC treated with ICI; *n* = 532	Mutation, any deleterious (*n* = 57, 11%) vs. WT	↔	11.0 vs. 12.4 0.83 (0.60–1.14); *p* = 0.25	
			Mutation, missense (*n* = 31, 6%) vs. WT	↔	11.9 vs. 12.4 1.03 (0.66–1.6); *p* = 0.87	
			Mutation, frameshift, nonsense, splice site (*n* = 26, 5%) vs. WT	↔	6.7 vs. 12.4 1.42 (0.92–2.2); *p* = 0.11	
		*KRAS*‐mutated metastatic NSCLC treated with ICI; *n* = 176	Mutation, any deleterious (*n* = 17, 10%) vs. WT	↓	3.0 vs. 15.1 0.29 (0.17–0.50); *p* < 0.001	
2021 Liu [29]	United States	*KRAS*‐mut LUAD treated with pembrolizumab [30] (MSK‐IO cohort); *n* = 77	Mutation, any (*n* = 9, 12%) vs. WT	↓	mPFS: 1.73 vs. 4.22 2.15 (1.46–4.35); *p* = 0.048	Not applicable
		*KRAS*‐mut LUAD treated with pembrolizumab (WFBCCC cohort); *n* = 18	Mutation, any (*n* = 2, 11%) vs. WT	↓	NS 11.98 (1.66–26.6); *p* = 0.0018	
		*KRAS*‐mut LUAD treated with non‐IO (TCGA cohort); *n* = 155	Mutation, any (*n* = 9, 6%) vs. WT	↓	15.7 vs. 19.7 2.32 (1.01–5.44); *p* = 0.047	
		*KRAS*‐mut LUAD treated with non‐IO (MSK‐CT cohort); *n* = 314	Mutation, any (*n* = 34, 11%) vs. WT	↓	5.2 vs. 6.5 1.95 (1.13–3.38); *p* = 0.015	
2020 Fernando [19]	United States	Stage 3B+ NSCLC (any treatment); *n* = 2462	Mutation, hm truncating (*n* = 102; 4%) vs. WT	↓	7.9 vs. 16.3^c^ 1.85 (1.47–2.34); *p* < 0.0001	Not applicable
			Mutation, hm non‐truncating (*n* = 101; 4%) vs. WT	↓	11.1 vs. 16.3^c^ 1.24 (0.97–1.58); *p* = 0.09	
			Mutation, ht truncating (*n* = 16; 0.6%) vs. WT	↔	11.6 vs. 16.3^c^ 1.19 (0.64–2.21); *p* = 0.59	
			Mutation, ht non‐truncating (*n* = 49; 2%) vs. WT	↔	19.8 vs. 16.3^c^ 0.93 (0.65–1.34); *p* = 0.70	
		Stage 3B+ NSCLC treated with ICI; *n* = 1176	Mutation, hm truncating (*n* = 38; 3%) vs. WT	↓	9.9 vs. 19.5^d^ 1.62 (1.12–2.36); *p* = 0.01	
			Mutation, hm non‐truncating (*n* = 41; 3%) vs. WT	↔	17.4 vs. 19.5^d^ 1.16 (0.80–1.67); *p* = 0.44	
			Mutation, ht truncating (*n* = 5; 0.4%) vs. WT	↔	39.0 vs. 19.5^d^ 0.84 (0.27–2.50); *p* = 0.76	
			Mutation, ht non‐truncating (*n* = 23; 2%) vs. WT	↔	27.0 vs. 19.5^d^ 0.79 (0.46–1.37); *p* = 0.40	
2020 Schoenfeld [14]	United States	NSCLC (any treatment); *n* = 1288	Mutation, class 1^e^ (*n* = 149; 12%) vs. WT	↓	NS; associated with worst outcomes	1.59 (1.25–2.04); *p* < 0.001^g^
			Mutation, class 2^f^ (*n* = 143; 11%) vs. WT	↓	NS	2.01 (1.58–2.55); *p* < 0.001^g^
		NSCLC treated with anti‐PD‐(L)1; *n* = 569	Mutation, class 1^e^ (*n* = 50, 9%) vs. WT	↔	NS^g^	Not applicable
			Mutation, class 2^f^ (*n* = 37; 7%)			
		*KRAS*‐mut NSCLC treated with anti‐PD‐(L)1; *n* = 374	Mutation, class 1^e^ (*n* = 58, 16%) vs. WT	↓	NS	1.59 (1.04–2.41); *p* < 0.001^g^
			Mutation, class 2^f^ (*n* = 52; 14%) vs. WT	↓	NS	2.75 (1.84–4.11); *p* < 0.001^g^

Abbreviations: adv, advanced; CI, confidence interval; CIT, chemoimmunotherapy; DFCI, Dana‐Farber Cancer Institute; hm, homozygous; HR, hazard ratio; ht, heterozygous; ICI, immune checkpoint inhibitor; LUAD, lung adenocarcinoma; LUSC, lung squamous cell carcinoma; m, median; MSKCC, Memorial Sloan Kettering Cancer Center; MSK‐CT, Memorial Sloan Kettering‐IMPACT Clinical Sequencing; MSK‐IO, Memorial Sloan Kettering‐immunotherapy; mut, mutated; NS, not specified; NSCLC, non‐small cell lung cancer; OS, overall survival; PFS, progression‐free survival; SMARCA4, SWItch/Sucrose Non‐Fermentable‐related BRG1/BRM‐associated factor chromatin remodeling complex subunit adenosine triphosphatase 4; VUS, variant of unknown significance; WFBCCC, Wake Forest Baptist Comprehensive Cancer Center; WT, wild‐type.

^a^ ↑: Significantly better prognosis. ↓: Significantly worse prognosis. ↔: No significant difference.

^b^ Most deletions were mono‐allelic.

^c^ *p* < 0.0001 for comparison of OS among the four mutation types and WT.

^d^ *p* = 0.093 for comparison of OS among the four mutation types and WT.

^e^ Truncating mutations, fusions, and hm deletions; 52% of *SMARCA4* variants.

^f^ Missense mutations or VUS; 48% of *SMARCA4* variants.

^g^ *p* < 0.001 for comparison among wild‐type, class 1, and class 2.

*SMARCA4* mutations are mutually exclusive with genomic alterations in other BAF genes profiled in the FoundationOne panel (*ARID1A*/*B*, *ARID2*, *PBRM1*, *SMARCB1*, *SMARCD1*), as well as with mutations in genes encoding prevalent targetable oncogenes, including *EGFR*, *ALK*, *ROS1*, *MET*, and *RET* (genomic profiling by Foundation Medicine Inc. [FMI]) [14, 16, 19]. A large proportion of *SMARCA4* gene alterations in NSCLC are homozygous, with over 40% of cases representing truncating mutations (class 1), suggesting LoF [19]. This is likely due to high rates of loss of heterozygosity (LOH) resulting in concomitant *KEAP1* and *STK11* genomic alterations, as all three genes are located in close proximity at chromosome 19p13.2–13.3 [14, 19]. The clinical relevance of this finding was illustrated in an analysis of a large database of 2462 patients treated in the Flatiron Health network who underwent routine comprehensive sequencing by FoundationOne or FoundationOne CDx. Patients with advanced (stage 3B or IV) NSCLC harboring homozygous truncating *SMARCA4* mutations had significantly worse median overall survival (mOS) compared to their wild‐type counterparts (7.9 vs. 16.3 months, hazard ratio [HR] 1.85) [19]. Similar results were observed among patients with homozygous mutations receiving immune checkpoint inhibitor (ICI) therapy (mOS 9.9 vs. 19.5 months, HR 1.62) [19]. However, a negative impact on survival was not observed among patients with heterozygous *SMARCA4* alterations [19]. Collectively, these data indicate that patients with advanced NSCLC harboring homozygous *SMARCA4* class 1 mutations represent a high‐risk patient population characterized by short overall survival, very low frequency of targetable mutations, and subpar outcomes following response to standard of care therapy with chemotherapeutic regimens or ICI.

Similar results were reported in an analysis of 4813 cases of advanced NSCLC treated at Memorial Sloan Kettering Cancer Center (MSKCC) who underwent genomic analysis by MSK‐IMPACT next‐generation sequencing (NGS). Multivariable analysis showed that SMARCA4 mutations were associated with significantly worse OS (*n* = 1288) [14]. Class 1 mutations were associated with the shortest OS (*p* < 0.001 vs. class 2 or wild‐type) [14]. Interestingly, patients with *SMARCA4*‐mutant tumors who received ICI therapy (*n* = 87) had better outcomes than those who did not (*n* = 205; HR for OS 0.67; 95% CI, 0.48–0.92; *p* = 0.01), particularly those with class 1 mutations (*p* value for overall response rate 0.027; *n* = 445) [14]. However, among patients who received ICI therapy, there was no difference in progression‐free survival (PFS) (*p* = 0.74) or OS (*p* = 0.35) based on whether *SMARCA4* was mutated or wild‐type (Table 1). *SMARCA4* alterations were more frequently observed with *KRAS*, *STK11*, and *KEAP1* mutations compared with SMARCA4 wild‐type counterparts [14].

Investigators at the Dana Farber Cancer Institute (DFCI) reported the outcomes of 1490 patients with metastatic NSCLC whose tumors were genetically profiled by targeted NGS focusing on the six BAF genes most often altered in NSCLC (*SMARCA4*, *ARID1A*, *ARID1B*, *ARID2*, *PBRM1*, and *SMARCB1*) [16]. BAF‐mutated NSCLC cases were more frequently associated with male sex, greater tobacco use, a higher tumor mutational burden (TMB), a higher proportion of advanced disease at diagnosis, and a lower proportion of targetable driver mutations compared to wild‐type cases [16, 31]. Compared with BAF wild‐type NSCLC, patients with BAF‐mutated NSCLC (*n* = 335) had a significantly shorter median OS from the time of advanced disease diagnosis (19.3 vs. 25 mos; HR 0.82; 95% confidence interval [CI] 0.71–0.96; *p* = 0.01), which was driven mainly by *SMARCA4*‐mutated cases (25 months vs. 15.6 months for *SMARCA4* wild‐type and mutated, respectively) [16]. Interestingly, among patients treated with ICI, no differences in clinical outcomes were observed between those with wild‐type and those with mutated *SMARCA4* alleles, with the exception of those with concurrent *KRAS* mutations (*n* = 176), where a *SMARCA4* mutation (*n* = 17) conferred a significantly lower overall response rate (ORR) (0% vs. 22%; *p* = 0.03), shorter median PFS (1.4 vs. 4.1 mos; HR 0.25; 95% CI 0.14–0.42; *p* < 0.001), and shorter mOS (3.0 vs. 15.1 mos; HR 0.29; 95% CI 0.17–0.50; *p* < 0.001) [16].

Mechanistically, mutated *SMARCA4*‐induced resistance to ICI has been linked to markedly decreased tumor infiltration of dendritic cells and CD4^+^ T cells and downregulation of STING, IL1β, and inflammatory cytokines required for efficient recruitment and activity of immune cells, secondary to loss of chromatin accessibility at enhancers of genes responsible for the innate immune response [32].

## 4 Genomic Context of SMARCA4 Alterations in NSCLC

A key modulator of the prognostic impact of *SMARCA4* alterations in NSCLC outcomes is the presence of other genomic alterations. While *SMARCA4* mutations are rarely identified in the presence of other BAF mutations or most targetable driver oncogenes [14, 16, 19], other alterations have been reported as highly prevalent in *SMARCA4*‐deficient NSCLC. In a study of 407 NSCLC cases harboring *SMARCA4* alterations, *TP53* (56%), *KEAP1* (41%), *STK11* (39%), and *KRAS* (36%) were frequently comutated [14]. *STK11*, *KEAP1*, and *SMARCA4* are tumor suppressors in lung tissue and mutations in those genes correlate with significantly worse outcomes for patients with NSCLC, particularly after ICI therapy [14, 16, 26, 27, 33, 34]. Given their tumor suppressive activities and the fact that all three co‐locate at chromosome 19p13.2–13.3, these genes are frequently found co‐deleted in NSCLC [16, 26, 33, 34]. Deletions of *STK11*, *KEAP1*, and *SMARCA4* alleles (most frequently monoallelic) were observed in 14.7%, 13.5%, and 13.7%, respectively among 3194 patients (2777 for *KEAP1* analysis) with non‐squamous NSCLC treated at MSKCC and DFCI [26]. The impact of haploinsufficiency of these genes on clinical outcomes was similar to that of mutations in these genes, and resulted in lower ORR and shorter PFS and OS among patients treated with chemotherapy or chemoimmunotherapy, independently of TMB or PD‐L1 expression [26]. In patients treated with ICI therapy alone, these deletions resulted in worse outcomes in the DFCI cohort among *KRAS*‐mutated cases, but had no effect among patients treated at MSKCC [26]. The reasons for this difference are unknown, but are likely related to differences in diagnostic assays, the heterogeneity of the treated populations, and the retrospective nature of these studies.

A large analysis of the impact of genomic and clinical features on the outcomes of 424 patients with *KRAS*‐mutated NSCLC identified comutations in the tumor suppressors *SMARCA4, KEAP1*, and *CDKN2A* as the most important independent determinants of inferior clinical outcomes with KRAS G12C inhibitor monotherapy (sotorasib or adagrasib) [28]. When analyzed individually, co‐mutations at the *SMARCA4*, *KEAP1*, or *CDKN2A* loci correlated with markedly shorter PFS and OS after treatment with sotorasib or adagrasib, and this deleterious effect was related to the number of comutations. Alterations at any of these three tumor suppressors identified approximately 50% of the patients with *KRAS*‐mutated NSCLC who experienced disease progression within 3 months from initiation of therapy [28]. It must be noted, however, that their impact as predictive markers of response was much less consistent [28]. It is also worth noting that patients with *KRAS*/*SMARCA4*‐comutated NSCLC have been shown to have a worse prognosis than those with only *SMARCA4*—and not *KRAS*—alterations [35]. While compelling, these results should be interpreted with caution as they are from retrospective uncontrolled studies. Ricciuti et al., partially addressed this issue by analyzing the genomes of 82 patients with NSCLC before ICI therapy and at the time of resistance to identify genomic lesions differentially acquired by tumors exposed to ICI [36]. At the time of acquired resistance, recurrent genomic changes (mutations and/or heterozygous loss) were observed in 62% of samples, and were coupled with decreased tumor‐infiltrating lymphocytes (TILs) and HLA class I expression in tumor biopsies (*n* = 8–16) [36]. The *B2M*, *SMARCA4*, *STK11*, and *KEAP1* loci were among the most frequently altered [36]. Control biopsies, from 138 patients treated with chemotherapy or targeted therapy as controls, did not exhibit these genomic and immunophenotypic changes [36]. These findings suggest that specific genomic lesions such as *SMARCA4* mutations are selected for during ICI therapy and likely play a key role in the development of ICI resistance. Incorporation of these findings may help refine prognostic tools so that they can more reliably stratify patients with NSCLC and maximize treatment outcomes.

## 5 Prognostic Relevance of ARID1A Mutations in NSCLC

ARID1A (AT‐interacting domain‐rich protein 1A) is the BAF subunit most frequently mutated in cancer and is a bona fide tumor suppressor [10]. *ARID1A* functions as a tumor suppressor gene in lung tissue and is mutated in approximately 8%–10% of patients with NSCLC, mainly as LoF alterations frequently associated with the loss of protein expression [13, 16, 37, 38]. Complete loss of or decreased ARID1A protein expression has been found to be significantly associated with LoF mutations and evidence of biallelic inactivation [38]. ARID1A recruits the BAF complex to target sequences via protein–DNA or protein–protein interactions [39]. ARID1A and its paralog ARID1B occupy the same position within the BAF complex and can functionally compensate for each other, which makes ARID1B essential to cancer cells following ARID1A mutation [40]. Downregulation of ARID1A has generally been reported to be an independent prognostic factor for shorter cancer‐specific survival in NSCLC [38, 41, 42] (Table 3).

**TABLE 3:** Studies evaluating ARID1A mutations or changes in expression in NSCLC.

Year, author	Country	Study design	Comparison	Prognostic impact^a^	mOS (mo) HR (95% CI); *p*‐value	Multivariable analysis HR (95% CI); *p*‐value
2023 Sun [43]	China	Stage IV LUAD harboring sensitive *EGFR* ^*MUT*^ treated with 1st‐gen EGFR TKIs; *n* = 57	Low (28; 49%) vs. high expression	↓	mPFS: 9.60 vs. 14.60; *p* = 0.0015 HR 0.45 (0.26–0.80)	Not applicable
2021 Alessi [16]	United States	Metastatic NSCLC (various treatments); *n* = 1490	Mutation, any deleterious (*n* = 105; 7%) vs. WT	↔	25.5 vs. 23.2 HR 1.13 (0.88–1.46); *p* = 0.34	Not applicable
2021 Zhu [13]	United States	NSCLC treated with ICI; *n* = 344	Mutation (*n* = 30; 9%) vs. WT	↔	21 vs. 11; *p* = 0.17 HR 0.709 (0.426–1.180)	Not applicable
2020 Hung [38]	United States	NSCLC (treatment NS); *n* = 2107	Mutation, any^b^ (*n* = 184, 8%) vs. WT	↔	NS; *p* > 0.05	Not applicable
		NSCLC (treatment NS); *n* = 139	Aberrant (*n* = 64, 46%) vs. intact protein expression^c^ by IHC in ARID1A‐mut tumors	↓	2.6 vs. not reached; *p* = 0.03 (*n* = 125)	
2020 Jang [41]	China	LUAD, LUSC treated with surgery; *n* = 171	Loss of (*n* = 23, 14%) vs. intact protein expression by IHC	↓	CSS: NS; *p* = 0.015	1.913 (1.095–3.342); *p* = 0.023
2020 Okamura [44]	United States	NSCLC treated with ICI; *n* = 375	Mutation (*n* = 46, 12%) vs. WT	↑	mPFS: 8.7 vs. 4.6; *p* = 0.53	Not applicable
2020 Wang [42]	China	NSCLC treated with surgery; *n* = 108	Reduced (*n* = 86; 80%) vs. increased protein expression by IHC	↓	NS; *p* < 0.001 5‐year OS: 54% vs. 79%	2.357 (1.315–4.895); *p* = 0.024

Abbreviations: ARID1A, AT‐rich interactive domain‐containing protein 1A; CI, confidence interval; CSS, cancer‐specific survival; EGFR, epidermal growth factor receptor; HR, hazard ratio; ICI, immune checkpoint inhibitor; IHC, immunohistochemistry; LUAD, lung adenocarcinoma; m, median; mut, mutant; NS, not specified; NSCLC, non‐small cell lung cancer; OS, overall survival; PFS, progression‐free survival; TKI, tyrosine kinase inhibitor; WT, wild‐type.

^a^ ↑: Significantly better prognosis. ↓: Significantly worse prognosis. ↔: No significant difference.

^b^ Loss‐of‐function mutations: *n* = 127 (69%), including nonsense mutations (*n* = 68, 37%), frameshift (*n* = 52, 28%), splice site (*n* = 6, 3%), structural rearrangement/truncating (*n* = 5, 3%). Missense mutations: *n* = 77 (42%).

^c^ Diffuse diminished expression: *n* = 17 (12%). Diffuse complete loss: *n* = 13 (9%). Intratumoral heterogeneous loss: *n* = 34 (25%).

Unlike *SMARCA4*, *ARID1A* is frequently comutated with *EGFR*, with the latter being mutated in 9% to 22% of NSCLC cases harboring *ARID1A* mutations [38, 45]. Importantly, *ARID1A* alterations have been associated with shorter PFS among patients with tyrosine kinase inhibitor (TKI)‐sensitive *EGFR*‐mutated NSCLC treated with first‐generation EGFR inhibitors [43]. A similar negative impact was observed in patients with *EGFR*‐mutated NSCLC treated with osimertinib or second‐generation EGFR inhibitors, in which *ARID1A* mutations were observed more frequently in tumors with *TP53* alterations [46]. Several mechanisms have been implicated in this insensitivity of *ARID1A*/*EGFR*‐comutated NSCLC to EGFR inhibitors, including the activation of compensatory signaling pathways (e.g., PI3K/Akt, JAK/STAT, and NF‐κB) and the promotion of epithelial to mesenchymal transition (EMT) and angiogenesis [47].

A large analysis of 29,757 FoundationCORE NSCLC samples found that *ARID1A* and *EGFR* were frequently comutated at diagnosis [37], indicating that ARID1A function is not critical for the survival of *EGFR*‐mutated NSCLC cells, and that *ARID1A* alterations may result in drug‐tolerant persister (DTP) phenotypes [48], which allow some NSCLC cells to survive EGFR TKI therapy, leading to clinical resistance. In *EGFR‐*mutated NSCLC cell lines, shRNA‐mediated knockdown of *ARID1A* promoted cell cycle activation, ErbB pathway activation, VEGF pathway activation, and expression of epithelial‐mesenchymal transformation (EMT) genes [43]. These findings suggest a multifactorial process through which ARID1A mutations increase tumor proliferation and metastasis, and decrease the sensitivity of *ARID1A*/*EGFR*‐comutated NSCLC to EGFR TKI therapy.

Outside of the *EGFR* comutation context, the opposite has been observed, with several studies reporting an association between *ARID1A* alterations and longer PFS and OS following treatment with ICI, not just in NSCLC but across a range of cancers [13, 44, 49]. Recent studies have shed light on this phenomenon. In a proteomic screen, ARID1A was shown to interact with mismatch repair (MMR) protein MSH2, which may explain its tumor suppressive role, as loss of ARID1A expression compromises MMR and increases mutagenesis and microsatellite instability. Increased mutagenesis results in a higher neoantigen load and TILs, making MMR‐deficient tumors more sensitive to ICI therapy [50, 51]. *ARID1A*‐deficient tumors are associated with high TMB and a more favorable prognosis in response to immunotherapy across multiple human cancers [13, 52]. In keeping with these findings, ARID1A expression has been found to be negatively correlated with TILs and PD‐L1 expression scores, used to predict the efficacy of treatment with ICIs [52], again linking loss of ARID1A function with increased sensitivity to ICI therapy.

A retrospective series involving 2440 consecutive patients with NSCLC highlights the danger of interpreting genomic data in the absence of protein expression correlates [38]. *ARID1A* mutations were detected in 7.5% of cases, of which 69% were LoF mutations [38]. ARID1A protein expression was aberrant in 46% of the 139 evaluable *ARID1A*‐mutated cases, with complete loss correlating with *ARID1A* premature‐truncating mutations and biallelic inactivation [38]. The concomitant presence of *ARID1A* mutations and aberrant ARID1A protein expression correlated with frequent *TP53* mutations and high TMB [38]. A separate study involving a cohort of 1013 NSCLC cases used for microarray analysis found that BAF subunit (ARID1A, SMARCA4, SMARCA2, and/or ARID1B) expression correlated with PD‐L1‐positive status and high TMB [31]. While patients with *ARID1A*‐mutated tumors exhibited similar OS as their wild‐type counterparts, the concomitant presence of *ARID1A* mutations and aberrant ARID1A expression correlated with shorter OS [38], which underscores the importance of interpreting *ARID1A* sequencing data in the context of the functional consequences of any specific mutation.

*ARID1A* alterations have also been linked to MMR deficiency and increased genomic instability. In a study that included 1540 patients and nine different tumor types, each with a prevalence of *ARID1A* alterations of at least 5%, the percentages of patients with microsatellite instability (MSI)‐high and TMB‐high (≥ 20 mutations/mb) were significantly higher in tumors harboring *ARID1A* alterations than in those with wild‐type *ARID1A* (20% vs. 0.9%; *p* < 0.001 and 26% vs. 8.4%; *p* < 0.001, respectively) [44]. This finding was also observed among the subset of 364 patients with NSCLC (5.9% vs. 0.4%; *p* = 0.01 [*n* = 267] and 26% vs. 8.6%; *p* = 0.03 [*n* = 345], respectively) [44]. *ARID1A* alterations were independently and significantly associated with longer PFS after ICI therapy across all histologies, including NSCLC, regardless of TMB and microsatellite status [44]. However, it must be noted that, while OS trended towards improvement among patients with *ARID1A* mutated tumors (vs. wild‐type), the difference was not statistically significant [44].

Overall, these data suggest that the presence of *ARID1A* alterations may predict sensitivity to ICI therapy across multiple tumor types. These observations merit the prospective validation of *ARID1A* alterations as a predictive and prognostic biomarker in patients with NSCLC, which could be helpful in refining patient stratification algorithms, along with more established markers such as PD‐L1 expression levels or TMB.

## 6 Prognostic Relevance of Mutations of Other BAF Subunits in NSCLC

Of the genomic alterations involving alleles encoding BAF subunits, those occurring at the *SMARCA4* and *ARID1A* loci have been the most thoroughly investigated in human cancer. Relatively little is known about the prognostic impact of genomic alterations of other BAF subunits in NSCLC. However, multiple subunits within the BAF complex have been described as having tumor suppressive activity and, not surprisingly, alleles harboring inactivating mutations have been reported across the spectrum of human cancer. For instance, *PBRM1*, *ARID2*, and *BRD7*, which encode subunits uniquely expressed by the pBAF complex, have been found to be mutated in approximately 1%–8% of human cancers [17, 53]. A genome‐scale CRISPR‐Cas9 screen to identify mechanisms of tumor cell resistance to killing by cytotoxic T cells identified the loss of over 100 genes, including *PBRM1*, *ARID2*, and *BRD7*, as sensitizing events to T cell‐mediated killing [54]. The pBAF complex has been shown to curtail chromatin accessibility to interferon (IFN)‐γ–inducible genes in cancer cells, thus promoting resistance to T cell‐mediated cytotoxicity. It follows, then, that *PBRM1*, *ARID2*, and *BRD7* genomic alterations that increase chromatin accessibility to IFN‐responsive genes may sensitize cancer cells to therapeutics that rely on T cell‐mediated cytotoxicity for their mechanism of action, including ICI, T cell engagers, and chimeric antigen receptor T cells [54].

This has been borne out in patients with metastatic clear cell renal cell carcinoma, a malignancy characterized by low TMB, a high frequency of *PBRM1*‐inactivating mutations (approximately 30%–41% of patients), and improved clinical responses to ICI therapy in the context of *PBRM1* mutations [53, 55, 56]. However, analyses in NSCLC have shown conflicting results. In a recent study, *PBRM1* mutations were detected in 84 of 2767 (3%) NSCLC cases, of which 60% were LoF mutations [57]. In spite of their association with higher TMB, *PBRM1*‐mutated tumors were linked to shorter OS among patients receiving ICI therapy, compared to their wild‐type counterparts [57]. *PBRM1* mutations did not appear to have a significant prognostic impact among patients with NSCLC treated with therapies other than ICI [57]. Similar conclusions were reached in a pan‐cancer analysis studying the impact of pBAF mutations on the outcomes of 2936 patients with 11 different tumor types receiving ICI therapy [53]. In most tumor types, *PBRM1* mutations, alone or in combination with *ARID2* mutations, were not significantly associated with OS even after adjusting for TMB [53]. However, in the NSCLC cohort, the presence of *PBRM1* and/or *ARID2* mutations was associated with statistically significant shorter OS after ICI therapy. Multivariable analysis showed that the presence of mutated *PBRM1* alleles was an independent predictor of worse OS in NSCLC (*n* = 983; HR 2.91; *p* < 0.001) after adjusting for TMB and copy number alterations [53].

While few studies have been published regarding the impact of *ARID2* mutations in NSCLC, it is worth noting that a composite analysis of five clinical cohorts treated with ICI at MSKCC (*n* = 2272) showed a numerical trend towards improved PFS (8.3 vs. 4.1 months, HR = 0.79, *p* = 0.4; *n* = 349) and OS (36 vs. 11 months, HR = 0.60, *p* = 0.097; *n* = 344) when comparing mutated to wild‐type *ARID2* cases, but the differences did not reach statistical significance [13].

In summary, while the available analyses are retrospective in nature and hindered by the limited number of mutated cases, the published data suggest that pBAF subunit alterations may be negative predictive biomarkers in NSCLC treated with ICI (Table 4).

**TABLE 4:** Studies evaluating *pBAF* mutations in NSCLC.

Year, author	Country	Study design	Comparison	Prognostic impact^a^	mOS (mo) HR (95% CI); *p*‐value	Multivariable analysis HR (95% CI); *p*‐value
***PBRM1***						
2020 Hakimi [53]	United States	NSCLC treated with ICI; 983	LOF mutation (*n* = NS) vs. WT	↓	NS	2.86 (1.72–4.74); *p* < 0.001
2020 Zhou [57]	United States	NSCLC treated with ICI; *n* = 412	Mutation (*n* = 24, 6%) vs. WT	↓	6 vs. 13; *p* = 0.03	2.16 (1.03–4.51); *p* = 0.041
		NSCLC treated with non‐ICI; *n* = 454	Mutation (*n* = 15, 3%) vs. WT	↓	NS; *p* = 0.048	Not applicable
***ARID2***						
2021 Zhu [13]	United States	NSCLC treated with ICI; *n* = 349	Mutation (*n* = 19; 5%) vs. WT	↔	mPFS: 8.3 vs. 4.1; *p* = 0.4 HR: 0.799 (0.474–1.346)	Not applicable
		NSCLC treated with ICI; *n* = 344	Mutation (*n* = 22; 6%) vs. WT	↔	36 vs. 11; *p* = 0.097 HR: 0.609 (0.331–1.117)	Not applicable

Abbreviations: CI, confidence interval; HR, hazard ratio; ICI, immune checkpoint inhibitor; LOF, loss of function; m, median; NS, not specified; NSCLC, non‐small cell lung cancer; OS, overall survival; pBAF, polybromo‐associated BRG1/BRM‐associated factor; PFS, progression‐free survival.

^a^ ↑: Significantly better prognosis. ↓: Significantly worse prognosis. ↔: No significant difference.

## 7 Conclusion and Future Directions

Our analysis of the subset of studies providing whole‐exome sequencing data on large numbers of patients with NSCLC shows that, overall, *SMARCA4* mutations are generally associated with poor prognosis regardless of therapy; *ARID1A* mutations are typically associated with better prognosis after ICI therapy; *ARD1A*/*EGFR*‐comutations are not susceptible to treatment with EGFR TKIs; and pBAF complex mutations, especially of *PBRM1*, are strongly associated with poor outcomes after ICI therapy. However, these conclusions were not universal across all the studies we reviewed. These discrepancies may reflect differences in patients' baseline characteristics, both between studies and between patients in individual studies.

To address this and allow more robust assessments of the role of BAF genomic lesions as predictors of therapeutic outcomes in NSCLC, several key factors will need to be considered, including the type of mutation (type 1 vs. type 2, homozygous vs. heterozygous), its functional consequences (complete, partial, or no protein expression), and the presence of comutations such as *STK11* and *KEAP1* both within and outside (e.g., other oncogenes or tumor suppressors) the BAF complex. In addition, PD‐L1 expression, TMB, MSI status, and density of TILs should be considered. Larger, functionally annotated, sufficiently powered, prospective clinical trials assessing uniformly treated patient populations are needed to validate these mutations as prognostic biomarkers and support their incorporation into treatment algorithms. With clinical trials of BAF complex‐directed therapies currently underway, it is tempting to speculate that some of these new therapeutics may ultimately reverse the deleterious impact of certain BAF genetic alterations.

## Author Contributions

**Alexis Khalil:** writing – original draft (supporting), writing – review and editing (equal). **Michael P. Collins:** writing – original draft (supporting), writing – review and editing (supporting). **Alfonso Quintás‐Cardama:** conceptualization (lead), supervision (lead), writing – original draft (lead), writing – review and editing (equal).

## Conflicts of Interest

A.K., A.Q.‐C., and M.P.C. are employees of Foghorn Therapeutics Inc.

## Data Availability

Data sharing not applicable to this article as no datasets were generated or analyzed during the current study.
